# Biomolecular Fingerprints of Sirtuin Activity in Senescent Fibroblasts Identified via Synchrotron-Based FTIR

**DOI:** 10.3390/ijms262110495

**Published:** 2025-10-29

**Authors:** Irene Fernández-Duran, Tanja Dučić, Alejandro Vaquero

**Affiliations:** 1 Chromatin Biology Laboratory, Josep Carreras Leukaemia Research Institute, 08916 Badalona, Spain; 2 ALBA-CELLS Synchrotron Light Source, 08290 Cerdanyola del Vallés, Spain

**Keywords:** sirtuins, cellular senescence, resveratrol, Fourier-transform infrared spectroscopy, genomic stability

## Abstract

Sirtuins are NAD^+^-dependent enzymes widely implicated in organismal ageing. In particular, nuclear-located sirtuins are histone deacetylases and/or monoADPrybosiltransferases that exert key roles in maintaining genomic stability. Although sirtuins have been reported to play an inhibitory role in cellular senescence, their specific targets and underlying mechanisms remain poorly understood. In this study, we use single-cell Synchrotron radiation-based Fourier-transform infrared spectroscopy (FTIR) to identify changes in biomolecular composition associated with cellular senescence induced by oxidative stress and replicative passaging in human primary fibroblasts. We also use the sirtuin activator resveratrol to determine which of these changes may be related to sirtuin activity. Resveratrol induced changes related to nuclear architecture, such as DNA conformation and nucleic acid–protein abundance ratios. Individual targeting of nuclear sirtuins was used to validate impaired DNA/protein ratios experimentally and provided a specific structural footprint associated with sirtuins in the context of cellular senescence. Altogether, this study reveals for the first time a sirtuin-dependent structural and biomolecular signature of senescence through single-cell FTIR, offering new insights into the cellular events underlying cellular senescence.

## 1. Introduction

Cellular senescence is a metabolically active cellular state characterized by cell cycle arrest, production of proinflammatory cytokines, and apoptosis resistance, among others [1]. Senescent cells accumulate during organismal ageing, and it has been proposed that this accumulation leads to the development of age-related diseases, from metabolic syndromes to neurodegenerative diseases [2]. At the biomolecular level, acquisition of a senescent phenotype is accompanied by a major reorganization of the nuclear compartment, which includes a prominent condensation of the nuclear DNA that promotes a repressive chromatin state (also known as senescence-associated heterochromatin foci or SAHF) [3]. Additionally, global proteomic and metabolomic rewiring are also hallmarks of senescent cells [1,4]. Although unique biomolecular profiles have been identified in age-related diseases such as amyotrophic lateral sclerosis [5], the cellular senescence biomolecular fingerprint, including simultaneous analysis of changes in DNA, protein, and lipid patterns, has not been well-defined. Considering the recent evidence suggesting a key causative role of cellular senescence in organismal ageing and age-related diseases [6,7], understanding the patterns of biomolecular alterations in senescence cells might give us key insights into the molecular alterations that occur during ageing and could provide novel universal markers of cellular senescence.

Sirtuins are a family of seven mammalian NAD^+^-dependent deacetylation enzymes that have been widely associated with cellular senescence and organismal ageing [8]. Recently, we have shown that skin cellular senescence can be prevented with the use of novel sirtuin modulators [9]. Amongst sirtuins, nuclear resident sirtuins (SIRT1, SIRT2, SIRT6, and SIRT7) play key epigenetic roles, from repression of gene expression through deacetylation of histone and non-histone proteins to structural control of the 3D chromatin architecture.

Diminished sirtuin levels or activity have been observed across numerous models of cellular senescence and organismal ageing. This decline contributes to impaired metabolic regulation, increased oxidative stress, and mitochondrial dysfunction—all hallmarks of ageing cells. Modulation of sirtuin activity has been suggested as a promising intervention to delay the onset of ageing. For example, supplementation with the metabolite NAD^+^—a cofactor for sirtuin deacetylase activity—or its precursors has been explored as a potentially beneficial strategy in ageing humans [10]. Moreover, resveratrol is a sirtuin activator commonly proposed as an anti-ageing substance [11].

In this study, we use single-cell Synchrotron radiation-based Fourier-transform infrared spectroscopy (SR-FTIR) to study global changes in biomolecular composition in cellular senescence, focusing on the potential implications of nuclear sirtuins in these changes. FTIR has previously been used as a valid approach to identify changes related to ageing or age-associated diseases [5,12]. Here, we use SR-FTIR to analyze concomitant biomolecular-associated spectroscopic changes at the single-cell level in senescent fibroblasts.

## 2. Results

### 2.1. FTIR Global Spectra of Human IMR90 Fibroblasts Undergoing Cellular Senescence

**Figure 1:**
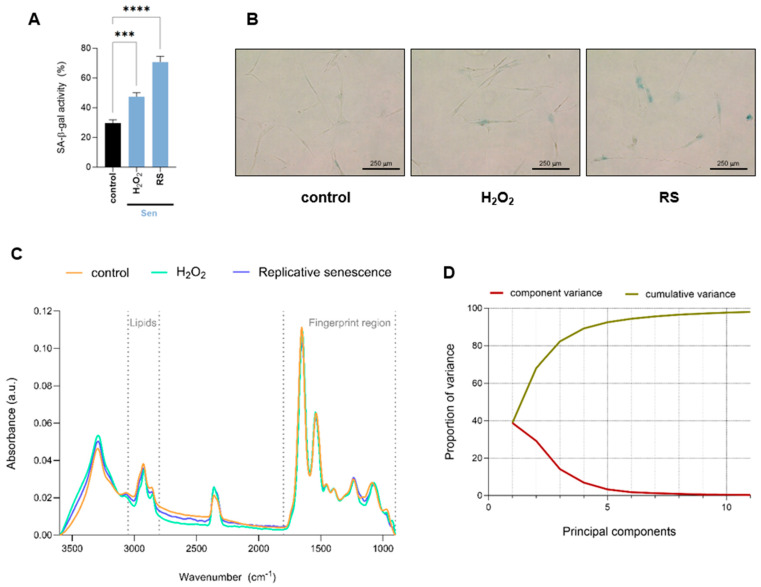
Overall biomolecular profiling of human fibroblasts undergoing cellular senescence by single-cell FTIR analysis. (**A**) Quantification of senescence-associated-β-galactosidase activity (SA-β-gal) of proliferative (control) and H_2_O_2_-treated (H_2_O_2_) or undergoing replicative senescence (RS) IMR90 human fibroblasts. Data represent the mean ± SEM of three biological replicates. (**B**) Representative pictures of cells quantified in (**A**). (**C**) Baseline-corrected and normalized spectra of the region 900–3600 cm^−1^ of proliferative (control) and senescent (H_2_O_2_-treated or undergoing RS) IMR90 human fibroblasts. The lipid (2800–3050 cm^−1^) and fingerprint (900–1800 cm^−1^) regions are marked by dashed lines. (**D**) Scree plot showing the proportion of variance explained (Y-axis) by each principal component (X-axis). The gradual decline indicates that multiple components are required to account for the total variance, reflecting the complexity of the dataset. a.u. = arbitrary units. *** *p* < 0.001, **** *p* < 0.0001.

Proliferating early passage human IMR90 fibroblasts were subjected to oxidative or replicative stress to induce cellular senescence by hydrogen peroxide (H_2_O_2_) treatment or serial passaging, respectively. Senescence-associated-β-galactosidase activity, one of the most commonly used markers of senescence, was measured to confirm cellular senescence induction. Under these conditions, both H_2_O_2_-treated and replicative senescent cells exhibited significantly increased levels of senescence-associated-β-galactosidase activity compared to control proliferating cells (Figure 1A,B). To gain a comprehensive understanding of biomolecular changes associated with the onset of senescence, proliferating fibroblasts (control), H_2_O_2_-treated fibroblasts (H_2_O_2_), and those undergoing replicative senescence (RS) were analyzed using single-cell FTIR. For each measurement, we selected areas where the cell nucleus was identified, so the spectra collected reflect both nuclear as well as cytosolic biomolecular composition. The complete baseline-corrected and normalized spectra of the region 900–3600 cm^−1^ are shown in Figure 1C. To further investigate the spectral differences among conditions, we performed multivariate analysis using Principal Component Analysis (PCA). The need for multiple principal components to explain the total variance suggests a relatively high level of spectral complexity, indicating that the variation among conditions is distributed across several features rather than dominated by a single source. In line with this, PC1 accounts for only 40% of the total variance, with the remaining variation spread across higher-order components (Figure 1D).

**Figure 2:**
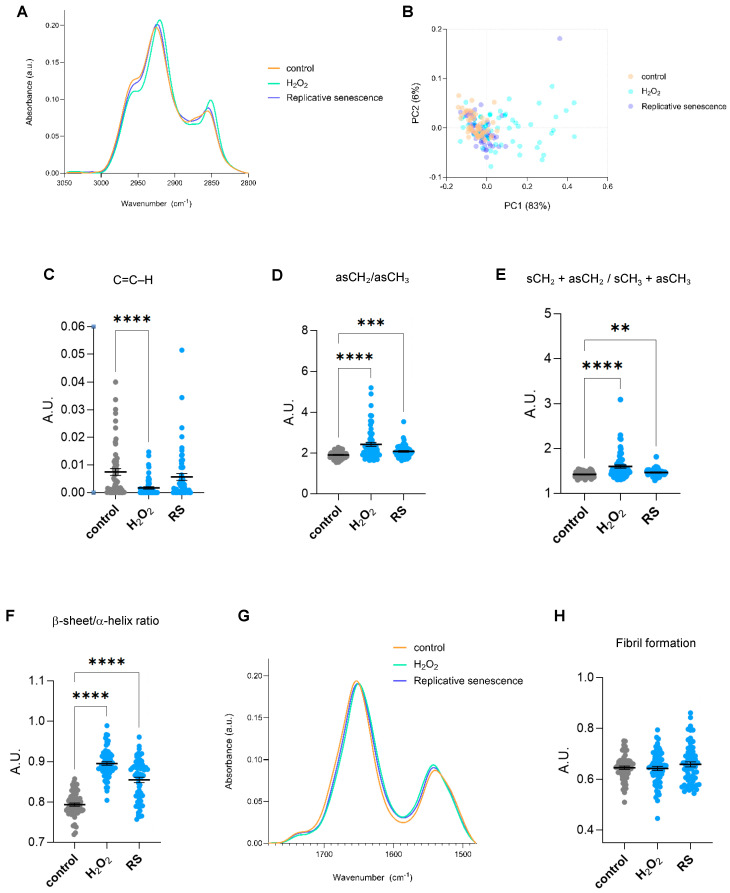
Lipid and proteomic biomolecular changes in human senescent fibroblasts by single-cell FTIR. (**A**) The normalized and averaged FTIR spectra of the lipid region 2800–3050 cm^−1^ of proliferative (control) and senescent (H_2_O_2_-treated (H_2_O_2_) or replicative-passaging (RS)) fibroblasts. (**B**) Principal Component Analysis (PCA) of the normalized and averaged FTIR spectra of the lipid region 2800–3050 cm^−1^. Each dot represents a single cell. (**C**) Integrated area under the C=C–H stretch peak at 3010 cm^−1^ (−/+5). The baseline-corrected and normalized spectra of the region 900–3600 cm^−1^ were used to calculate date in (**B**–**E**). (**D**) Integrated area of the asymmetric CH_2_/CH_3_ stretching regions (2945–3000 and 2900–2945 cm^−1^ bands). (**E**) Integrated area of the total CH_2_ (sCH_2_ + asCH_2_) divided by the total CH_3_ (sCH_3_ + asCH_3_) stretching regions. (**F**) Ratio of the integrated area of β-sheet/α-helix secondary protein structural conformation, calculated as the integrated areas of 1630–1640 and 1650–1660 cm^−1^ bands, respectively. (**G**) The averaged FTIR spectra of the region 1480–1780 cm^−1^ of proliferative (control) and senescent (H_2_O_2_-treated or replicative-passaging) fibroblasts. (**H**) Fibril formation calculated using the ratio of Amide II/Amide I intensities. Amide II and Amide I peaks were defined as the regions spanning 1480–1580 and 1600–1700 cm^−1^, respectively. The respective integrated areas were calculated after baseline correction and normalization of the fingerprint area (900–1800) cm^−1^. a.u. = arbitrary units. ** *p* < 0.01, *** *p* < 0.001, **** *p* < 0.0001.

Different FTIR regions of the spectrum give information about distinct biomolecules. The spectrum ranging 2800–3050 cm^−1^ offers insights regarding the lipid content, whereas the 900–1800 cm^−1^ region, also known as the fingerprint area, contains bands majorly related to components of proteins, nucleic acids, and carbohydrates. Senescent cells undergo significant lipid remodelling, characterized by the accumulation of lipids in structures such as lipid droplets, alongside changes in lipid composition that alter membrane properties [13]. Single-cell SR-FTIR analysis of the 2800–3050 cm^−1^ spectral area revealed changes in lipid composition in cells undergoing cellular senescence, especially in H_2_O_2_-treated cells (Figure 2A). Multivariate statistical analysis was applied to assess the spectral differences across experimental conditions. Specifically, Principal Component Analysis (PCA), was used to evaluate clustering and separation between groups. The results show that the first principal component (PC1) accounts for the largest proportion of variance (83%) (Figure 2B). H_2_O_2_-treated cells appear most distinct from both proliferative and replicative senescence cells along PC1, indicating significant spectral differences. The band at ~3010 cm^−1^ has been associated with =CH stretching vibrations of unsaturated (C=C–H) lipids [14]. Cells treated with H_2_O_2_ presented significantly lower levels of the integrated area at ~3010 cm^−1^ (Figure 2C), suggesting less unsaturated bonds. However, since this observation was only present in cells treated with H_2_O_2_ but not subjected to replicative senescence, it might reflect a direct effect of oxidative stress but not a cellular senescent-specific feature. In this region, symmetric and asymmetric vibrations of CH_2_ and CH_3_ groups of lipids can also be assigned to peaks in the 2800–3000 cm^−1^ region. Changes in fatty acid chain length or membrane order were inferred from FTIR by integrating the ratio of asymmetric CH_2_ and CH_3_ (*ν_as_*CH_2_/*ν_as_*CH_3_) stretching regions (2900–2945 and 2945–3000 cm^−1^, respectively). Increased *ν_as_*CH_2_/*ν_as_*CH_3_ ratios were observed in senescent cells, indicating alterations in lipid structure and saturation (Figure 2D). Moreover, changes in acyl chain length were assessed as the ratios between total CH_2_ and total CH_3_ peak intensities. Senescent cells displayed significantly higher levels of CH_2_/CH_3_ ratios, indicating longer fatty acid chains or increased chain ordering (Figure 2E). H_2_O_2_ treatment also showed a shift in *ν_as_*CH_2_ position in comparison to control and replicative senescent cells (Figure 2A). Since the precise wavenumber position of the asymmetric CH_2_ stretching absorption band is an indicator of the conformational flexibility and dynamic behaviour of phospholipid acyl chains [14,15], this observation implies changes in the fluidity and normal functioning directly linked to an increased oxidative state. Altogether, we identified changes in fatty acid length and saturation that might reflect the known alterations occurring in senescent cells, including lipid composition (e.g., more saturated fatty acids, less cholesterol), and accumulation of lipid rafts or ceramides. Next, we focused on the fingerprint region. Previous reports have identified changes in α-helix/β-sheet conformation ratio and fibril formation in aged or UV-treated human dermal fibroblasts [6,16]. A second derivative of spectra of the Amide I/Amide II region (1480–1780 cm^−1^) and the identified seven minor peaks were used for the Amide I/Amide II peak fitting to analyze potential changes in specific secondary structures. Significant changes were observed at subpeaks associated with bands near 1655, 1635, 1681, and 1570 cm^−1^. Calculation of the integrated area of the subpeaks at 1655 and 1635 was used to measure β-sheet to α-helix secondary structure ratio levels. Senescent cells displayed higher levels of β-sheet to α-helix ratio (Figure 2F), in line with previous observations in UV-irradiated dermal fibroblasts [6]. The Amide I/Amide II region was also used to analyze potential changes in fibril formation. No significant changes were found in senescent cells regarding fibril structures (analyzed as the ratio of the integrated area of Amide I/Amide II) (Figure 2G,H).

### 2.2. Genomic and Epigenetic Profiling by FTIR of Human IMR90 Fibroblasts Undergoing Cellular Senescence

**Figure 3:**
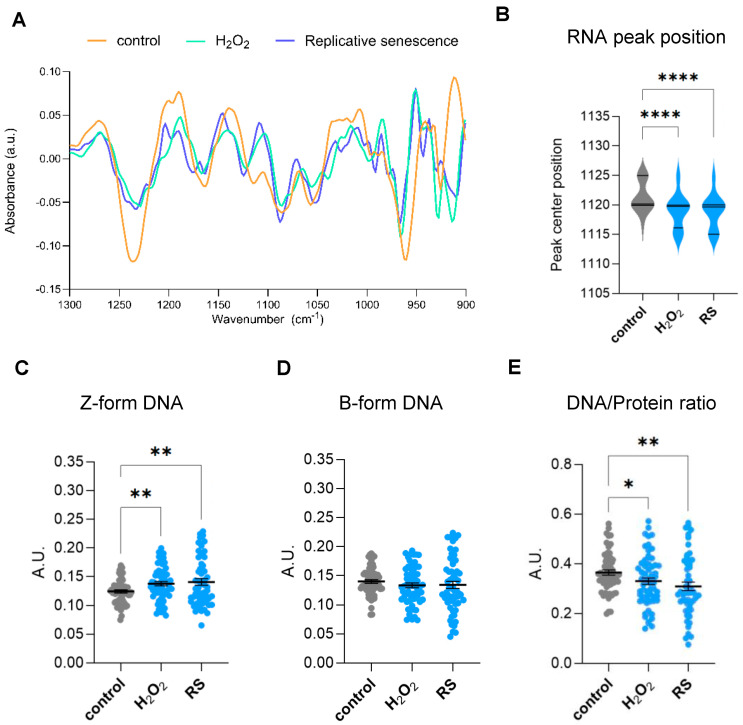
Analysis of genomic and epigenomic-associated FTIR bands. (**A**) Spectra obtained from the Savitzky–Golay second derivative spectra of the 900–1300 cm^−1^ area of proliferative (control) and senescent (H_2_O_2_-treated (H_2_O_2_) or replicative-passaging (RS)) fibroblasts. The second derivative was calculated using a Savitzky–Golay filter with a window size of 21 points and a polynomial order of 3 and normalized through vector normalization. (**B**) Analysis of peak centre position of the RNA peak band (1120 cm^−1^) after a delta-fixed (±5) centre peak fitting using a Gaussian model of the Savitzky–Golay second derivative spectra 900–1300 cm^−1^ area. (**C**) Integrated area under the Z-form peak at 1059 cm^−1^ (±3) after baseline correction and normalization of the fingerprint area (900–1800) cm^−1^. (**D**) Integrated area under the B-DNA form peak at 1089 cm^−1^ (±3) after baseline correction and normalization of the fingerprint area (900–1800) cm^−1^. (**E**) Single-cell DNA/protein ratio calculated as the ratio between the DNA content (as the integrated 1070–1135 cm^−1^ area) and protein content (as the integrated Amide II 1490–1580 cm^−1^ area), both calculated from the baseline-corrected and normalized spectra within the region 900–3600 cm^−1^. a.u. = arbitrary units. * *p* < 0.05, ** *p* < 0.01, **** *p* < 0.0001.

The spectral range covering a specific region of the fingerprint area (900–1800 cm^−1^) is highly intricate because of different biomolecules that have closely located bands that arise in this zone. In particular, some of these bands in the 900–1300 cm^−1^ region are associated with carbohydrates and phosphates, which are backbone molecules of nucleic acids. Therefore, genomic information can be retrieved from specific bands in this range. Moreover, several bands appearing in this complex region have also been associated with epigenetic effects, including methylation and acetylation reactions [17,18]. A second derivate of the area from 900 to 1300 cm^−1^ identified subpeaks at 968, 1059, 1089, 1115, 1089, 1160, and 1240 cm^−1^, as shown in Figure 3A. The peak at 968 cm^−1^ is known as a DNA marker [16]. The band at 1059 cm^−1^ has been described to be associated with a strong form of Z-DNA, whereas the band at 1089 cm^−1^ to B-form conformed DNA as well as *ν*PO4^2−^ of phosphorylated proteins [17]. Absorptions at 1115 cm^−1^ have been associated with vibrations of symmetric stretching of the phosphodiester (PO_2_^−^) group of RNA backbone [19], whereas absorptions at 1220 cm^−1^ have been previously linked to DNA-specific backbone vibrations [20]. Subtle shifts in the ~1115 cm^−1^ peak might reflect structural changes, such as strand breaks or base modifications, that can modify the phosphate backbone vibrations. Interestingly, we observed a prominent change in the peak position at 1115 cm^−1^, suggesting changes in the RNA environment in senescent cells (Figure 3B). Previous studies have linked acetylation to DNA conformational structure. In particular, Zhang et al. showed by FTIR that addition of trichostatin A (TSA), a reversible inhibitor of histone deacetylases (HDACs), promotes transformation of B-DNA to Z-DNA in cells [17]. Quantification of bands at 1059 cm^−1^ (±3) and 1089 cm^−1^ (±3) peaks showed a clear increase in Z-form DNA conformation while no significant changes were observed in B-form DNA (Figure 3C,D). Nucleosome repositioning or histone modifications may increase negative supercoiling, facilitating B-Z DNA transitions at regulatory sites. However, since B-form DNA amounts are not altered in senescent cells (Figure 3D), the increase in Z-DNA is more likely caused by either an increase in cytosine methylation or activity of Z-DNA binding proteins, as both processes can promote Z-DNA stabilization.

During the acquisition of a senescent phenotype, cells also experience major morphologic changes. In vitro senescent fibroblasts show a characteristic flattened morphology and increased cell size due to cytoskeletal reorganization, nuclear architecture changes, and increased protein synthesis, among others [1,2]. Therefore, we decided to investigate changes in the biomolecular composition of senescent cells by analyzing the ratios between DNA and protein levels obtained by FTIR at the single-cell level. To do so, we analyzed single-cell content DNA/protein ratios, considering the integrated 1070–1135 cm^−1^ area as a measurement of total DNA content and the integrated Amide II 1490–1580 cm^−1^ area as the measurement of total protein content. This analysis showed that fibroblasts undergoing oxidative-stress- or replicative-stress-induced senescence display significantly lower levels of DNA/protein ratios per cell (Figure 3E).

### 2.3. Effect of Resveratrol on the Biomolecular Composition and Structure of Fibroblasts Undergoing Replicative Senescence Analyzed by Single-Cell FTIR

**Figure 4:**
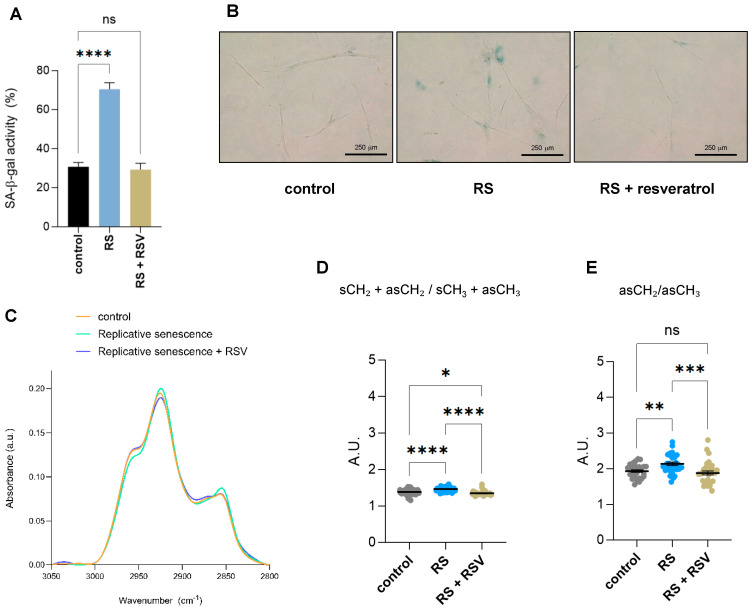
Effect of resveratrol on lipid composition in replicative senescent IMR90 fibroblasts Analyzed by single-cell FTIR. (**A**) Senescence-associated-β-galactosidase activity (SA-β-gal) staining of IMR90 fibroblasts either proliferating (control), undergoing replicative senescence (RS) or treated with 50 mM resveratrol for 48 h while undergoing replicative senescence (RS + RSV). Data represent the mean ± SEM of three biological replicates. (**B**) Representative pictures of cells quantified in (A). (**C**) The normalized and averaged FTIR spectra of the lipid region 2800–3050 cm^−1^ of proliferative (control), senescent (RS) or senescent fibroblasts treated with 50 mM resveratrol for 48 h (RS + RSV). (**D**) Integrated area of the total CH2 (sCH_2_ + asCH_2_) divided by the total CH3 (sCH_3_ + asCH_3_) stretching regions. The baseline-corrected and normalized spectra of the region 900–3600 cm^−1^ were used to calculate date in (**D**,**E**). (**E**) Integrated area of the asCH_2_/asCH_3_ stretching regions (2945–3000 and 2900–2945 cm^−1^ bands). a.u. = arbitrary units. * *p* < 0.05, ** *p* < 0.01, *** *p* < 0.001, **** *p* < 0.0001, ns: not significant.

To understand which of the observed changes by single-cell FTIR were due to sirtuin activity, we treated primary IMR90 fibroblasts undergoing replicative senescence with the sirtuin activator resveratrol and subjected them together with early passage proliferating and replicative senescent IMR90 fibroblasts to single-cell FTIR analysis. Addition of resveratrol reduced the senescence-associated-β-galactosidase activity of fibroblasts undergoing replicative senescence due to serial passaging (Figure 4A,B). Changes in biomolecular composition upon the addition of resveratrol to replicative senescent fibroblasts were Analyzed by single-cell FTIR. Analysis of the FTIR spectra of the lipid region 2800–3050 cm^−1^ showed that the lipid composition of cells undergoing replicative senescence treated with resveratrol resemble control proliferative fibroblasts (Figure 4C). Next, we analyzed the acyl chain length and/or degree of chain branching using the symmetric and asymmetric CH_2_ and CH_3_ bands, as we had found two derived parameters significantly deregulated in senescent cells (Figure 2D,E). Addition of resveratrol had a clear impact on the two lipid structural markers, (sCH_2_ + asCH_2_/sCH_3_ + asCH_3_ and asCH_2_/asCH_3_ ratios), as both displayed significantly lower levels after resveratrol treatment in replicative senescent cells, reaching similar levels to control cells (Figure 4D,E). Altogether, these results suggest that some of the alterations in lipid conformation in senescent cells are regulated by sirtuins, as their activation can revert this effect.

**Figure 5:**
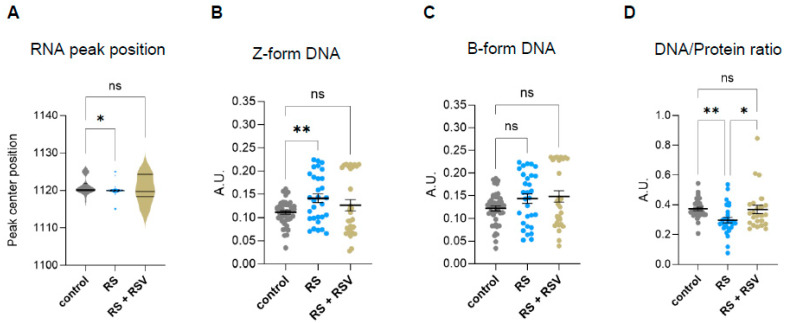
Effect of resveratrol on DNA conformation and nucleic acid/protein ratios in replicative senescent IMR90 fibroblasts Analyzed by single-cell FTIR. (**A**) Analysis of peak centre position of the RNA peak band (1120) after peak fit of the Savitzky–Golay second derivative spectra 900–1300 cm^−1^ area. (**B**) Integrated area under the Z-form peak at 1059 cm^−1^ (−/+3) after baseline correction and normalization of the fingerprint area (900–1800) cm^−1^. (**C**) Integrated area under the B-DNA form peak at 1089 cm^−1^ (−/+3) after baseline correction and normalization of the fingerprint area (900–1800) cm^−1^. (**D**) Single-cell DNA/protein ratio calculated as the ratio between the DNA content (as the integrated 1070–1135 cm^−1^ area) and protein content (as the integrated Amide II 1490–1580 cm^−1^ area), both calculated from the baseline-corrected and normalized spectra within the region 900–3600 cm^−1^. * *p* < 0.05, ** *p* < 0.01, ns: not significant.

Sirtuins are well-known regulators of chromatin structure, nuclear architecture, and epigenetics. Thus, we next investigated the effect of resveratrol on the changes in nucleic acid conformation that we had previously identified in senescent cells by FTIR in the fingerprint area (Figure 3B–D). Analysis of specific RNA peak position in resveratrol addition in replicative senescence cells revealed no significant changes to proliferating cells (Figure 5A), suggesting sirtuin-mediated regulation of the RNA molecule environment in senescent cells. Moreover, resveratrol reduced Z-DNA content in senescent fibroblasts while not impacting B-DNA (Figure 5B,C). These results suggest that the increase in Z-DNA content in senescent cells might be regulated by one or more sirtuins.

We then explored whether the single-cell DNA/protein ratios were also affected by sirtuin activity. Addition of resveratrol recovered the decreased DNA/protein ratios observed in senescent fibroblasts, suggesting that sirtuins are required to prevent deregulated DNA/protein ratios in senescent cells (Figure 5D).

### 2.4. Targeting Nuclear Sirtuins Impairs Cellular DNA/Protein Ratios

**Figure 6:**
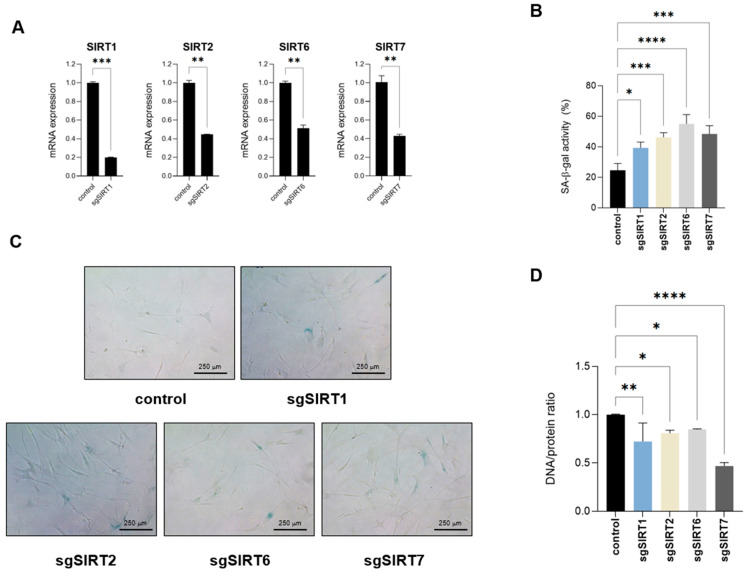
Targeting nuclear sirtuins impairs DNA/protein ratios. (**A**) Relative mRNA expression levels of SIRT1, 2, 6 or 7 were quantified by RT-qPCR in IMR90 fibroblasts following stable knockdown using CRISPRi, compared to control cells. Data represent the mean ± SEM of three biological replicates. (**B**) Quantification of senescence-associated-β-galactosidase activity (SA-β-gal) of control or sirtuin-targeted IMR90 fibroblasts. Data represent the mean ± SEM of three biological replicates. (**C**) Representative pictures of cells quantified in (**B**). (**D**) Genomic DNA and total protein content was extracted from control or IMR90 fibroblasts with downregulated SIRT1, SIRT2, SIRT6 or SIRT7 expression. Genomic DNA/protein ratios are shown. Data represent the mean ± SEM of three biological replicates. * *p* < 0.05, ** *p* < 0.01, *** *p* < 0.001, **** *p* < 0.0001.

Since the addition of resveratrol was able to revert several biomolecular composition and structural changes occurring in replicative senescent fibroblasts, including those affecting genomic structure and DNA/protein composition, we decided to target each nuclear sirtuin individually and assess their potential effect on acquiring a senescent phenotype. Because sirtuins play a fundamental role in DNA repair, we used a CRISPR interference (CRISPRi) approach, silencing specific sirtuin expression without cutting genomic DNA [21]. Using this system, we were able to generate IMR90 fibroblasts with targeted sirtuin downregulated expression of at least 50% compared to control cells (Figure 6A). Analysis of these cells showed an increase in SA-β-galactosidase activity upon silencing of any of the four sirtuins (Figure 6B,C). Among the different biomarkers that we have identified by FTIR and that were abrogated by resveratrol and thus potentially linked to sirtuin function, DNA/protein rations can easily be measured. Therefore, we Analyzed potential changes in the DNA/protein ratios upon sirtuin downregulation. We extracted genomic DNA and total protein content of each sample and normalized for the same number of cells. Analysis of the DNA/protein samples of these samples showed a decrease in the DNA/protein ratios upon individual nuclear sirtuin silencing (Figure 6D), further confirming the anti-senescent impact of sirtuin function. Remarkably, SIRT7 was the sirtuin that had a major impact on the DNA/protein ratio. Considering that all four sirtuins promoted a similar increase in SA-β-galactosidase activity (Figure 6B), this suggests that SIRT7 may have an additional impact in cell senescence in contrast to the rest of sirtuins. Overall, these results suggest that DNA/protein ratios are altered in senescent fibroblast cells in a sirtuin-dependent manner and may serve as a marker of cellular senescence. This alteration may represent a potential novel biomarker for identifying senescent fibroblasts.

## 3. Discussion

Using single-cell SR-FTIR, we have identified several changes in biomolecular composition and structure in human fibroblasts undergoing cellular senescence. We identified changes in lipid composition, protein structure, and DNA architecture in fibroblasts subjected to oxidative or replicative stress. Since lack of a universal marker that identifies senescent cells remains a challenge in the field [22], validation of the different biomarkers that we have identified to be altered in human fibroblasts undergoing cellular senescence due to oxidative or replicative stress remain to be validated in other cellular models of senescence and ageing. This is of particular interest for certain models or experimental settings where a limited sample is available or single-cell analysis is preferential given that SR-FTIR allows for non-destructive analysis with minimal sample preparation.

The PCA results revealed that PC1 accounted for only 40% of the total variance, with multiple principal components required to capture the remaining spectral variation. This distribution of variance suggests a high degree of complexity and heterogeneity within the senescent cell population. Such complexity likely reflects the diverse biochemical and structural changes that occur during senescence, highlighting that senescent cells exhibit multifaceted alterations rather than a uniform phenotype. These findings underscore the importance of using comprehensive multivariate approaches to fully characterize the senescence-associated spectral signatures.

Regarding changes in lipid composition, we observed an increase in total CH_2_ vs. CH_3_ composition as well as in the asymmetric CH_2_ vs. CH_3_ ratio (*ν_as_*CH_2_/*ν_as_*CH_3_) in senescent cells, altogether implying longer, more ordered or increased saturated fatty acid chains, potentially suggesting reduced membrane fluidity. Senescent cells and ageing organisms exhibit higher levels of saturated fatty acids which have been shown to promote inflammatory pathways [23,24]. Fatty acid metabolism is highly regulated by sirtuin function: mitochondrial sirtuins directly modify key enzymes involved in fatty acid β-oxidation and energy production whereas nuclear sirtuins regulate fatty acid metabolism by controlling transcriptional programmes and signalling pathways, including PPARα, PGC-1α, and AMPK, which coordinate the expression of genes involved in lipid uptake, oxidation, and mitochondrial biogenesis. In agreement with the well-established role of sirtuins as lipid metabolism regulators, we observed that the addition of resveratrol reverted the increase in these ratios in cells undergoing replicative senescence. Additionally, we suggest that the identified lipid-related FTIR features in senescent cells are promising candidates for in vivo senescence markers in age-related diseases where oxidative stress is a major initiating factor or is directly linked to senescence. Such diseases include Alzheimer’s disease, atherosclerosis, and idiopathic pulmonary fibrosis, in which oxidative stress plays a central role in triggering or sustaining the senescent phenotype.

We also observed senescence-associated changes in the fingerprint area related to protein and nucleic acid structures. In agreement with previous reports [6], higher levels of β-sheet-to-α-helix protein conformation ratio were observed in senescent fibroblasts. Increased β-sheet content in senescent cells has been linked to accumulation of misfolded or aggregated proteins [25]. We did not observe changes in fibril formation in senescent cells by FTIR. Since fibril formation is a common feature of some neurodegenerative diseases, whether changes in fibrillar protein conformation might occur in senescent cells should be explored in other cell identities such as in the neuronal context.

FTIR analysis also revealed nucleic acid conformational modifications in senescent cells. Interestingly, we observed changes in the RNA peak position, which were reversed by resveratrol, suggesting sirtuin-mediated control of RNA structures. The relationship between sirtuins and RNA structural changes is not fully understood, although growing evidence suggests that sirtuins can indirectly and, in some cases, directly influence RNA structure, stability, and function through several mechanisms. For example, some nuclear sirtuins can regulate the expression of RNA-binding proteins and RNA-modifying enzymes, which in turn influence RNA structure and function [26]. Moreover, SIRT7 directly binds RNA [27], although the physiological implications of this remain to be determined.

By using SR-FTIR, we also detected an increase in Z-DNA formation in senescent cells. Importantly, Z-DNA has been associated with human diseases through recruitment of DNA binding proteins, transcriptional regulation, or induction of genome instability, among others [28]. In fact, Z—DNA binding protein 1 (ZBP-1) regulates critical pathways associated with cellular senescence and ageing, including DNA repair, inflammation, and dysfunctional mitochondrial signalling [29]. Nonetheless, previous studies have linked acetylation to DNA conformational structure. In particular, Zhang et al. showed by FTIR that the addition of trichostatin A (TSA), a reversible inhibitor of histone deacetylases (HDACs), promotes transformation of B-DNA to Z-DNA in cells [17]. Since Z-DNA is involved in the regulation of gene expression, genome stability, and innate immune activation, processes that are also known to be extensively regulated by sirtuins, whether this family of proteins are involved in Z-DNA regulation directly or indirectly remains to be explored. Nonetheless, SR-FTIR analysis of Z-DNA levels might be of specific relevance, for instance, as prognosis markers, in some diseases where Z-DNA metabolism is altered. For example, a recent report demonstrates increased Z-DNA accumulation in cutaneous lupus erythematosus keratinocytes in response to autoimmune photosensitivity [30]. Moreover, another recent investigation showed that amyloid-β induces Z-form conformation in Alzheimer’s Diseases microglia [31]. Understanding whether this accumulation also occurs in the microglia present in other neurodegenerative diseases might be particularly important, given the crucial role of this cell type in neuroinflammation and disease progression.

Changes in cell size and shape are a well-established biomarker of cellular senescence. Indeed, increased cell size is not only a characteristic of senescent cells but can also be causative of the senescent phenotype by itself [32]. Aberrant cell growth in senescent cells promotes cytoplasmic dilution, which in turn perturbs the DNA/cytoplasm ratio contributing to cellular senescence [33]. De Cecco et al. used single-cell resolution fluorescence microscopy to show that in human dermal and mouse tail fibroblasts, while total DNA content remained unperturbed, nuclear protein accumulation occurred [34]. Nonetheless, increased synthesis of some cytoplasmic proteins also occurs in senescent cells, for example, to promote and sustain the senescence-associated secretory phenotype (SASP) [35]. Moreover, accumulation of misfolded proteins has been described in organismal ageing [36]. In fact, treatment with rapamycin, which targets protein synthesis, has been suggested as a therapeutic strategy to target senescent cells and, in particular, the SASP [37,38]. In contrast, since senescence cells do not divide, their DNA content remains stable. Our results show decreased DNA/protein ratios in H_2_O_2_-induced and replicative senescent fibroblasts, which in turn can be reverted by resveratrol treatment in the later. This is in line with previous studies that have shown by FTIR that treatment of B cells with TSA diminishes the DNA/protein ratio of cells [39]. Together, these results suggest that modulation of acetylase activity impacts cellular DNA/protein ratios in a reversible manner. Nonetheless, we have observed that targeting individually nuclear sirtuins also diminishes the cellular DNA/protein ratio. How nuclear sirtuins diminish DNA/protein ratios remains to be investigated. Sirtuins regulate cellular senescence through many different roles, some of which imply loss of proteostasis mechanisms. Hence, it is plausible that one of the reasons sustaining the altered DNA/protein ratios is related to the role of sirtuins in maintaining cellular proteostasis. In senescent cells, downregulation of sirtuins levels or activity may lead to impaired chaperone induction, defective autophagy and mitophagy, accumulation of damaged proteins and enhanced SASP, and proinflammatory signalling. Of interest, among the nuclear sirtuins, we observed a pronounced effect with SIRT7. Compared to the other nuclear sirtuins, SIRT7 is also present in nucleolus [40]. Whether this specific localization might impact overall genomic DNA or protein content in a unique manner in senescent cells remains to be further investigated. Altogether, we propose several novel SR-FTIR-based senescent markers using biomolecular fingerprints of single cells.

The high-end SR-FTIR resolution used in this study allows for the identification of the nucleus and the cytoplasm of each cell. However, small organelles such as mitochondria remain beyond SR-FTIR’s current ability to resolve. Given the complexity of biomolecular composition at the subcellular level, spatial resolution of intracellular components will help improve measurement consistency between cell and cell acquisition of the same population. Recent advancements in optical development are improving the spatial resolution limits of SR-FTIR. For example, combination of near-field optical microscopy with SR-FTIR can provide biomolecular profiles at the subcellular scale [41], suggesting that further optical innovations will allow for greater specific subcellular deconvolution. Alternatively, SR-FTIR can be combined with fluorescence microscopy to correlate FTIR spectra to specific structures that can be stained using fluorescent probes. Using this approach, Álvarez-Marimón et al. recently showed lipid oxidation alterations detected by FTIR at non-fibrillary plaques in brain tissue samples from Alzheimer’s disease patients [42]. Combining SR-FTIR with novel optical approaches or fluorescence strategies will improve subcellular precision and, given the inherent high biomolecular complexity of cells, favour the identification of novel FTIR cellular senescence markers.

## 4. Materials and Methods

### 4.1. Cell Culture and Treatments

Human IMR90 fibroblasts were obtained from ATCC (Manassas, VA, USA). IMR90 and HEK293T cells were cultured in Dulbecco’s modified eagle medium supplemented with 10% fetal bovine serum and 10 U/mL penicillin/streptomycin in a humidified incubator at 37 °C and 5% CO_2_. For cell culture, cells at 80% confluence were trypsinized and plated at 60% confluence. Proliferating or replicative senescent cells were treated with 100 μM H_2_O_2_ or 50 μM resveratrol for 48 h, respectively.

### 4.2. Senescence-Associated-β-Galactosidase Staining

For senescence-associated-β-galactosidase staining, equal amounts of cells were seeded on 6-well plates. Cells were washed three times with PBS 1× and fixed with 0.5% glutaraldehyde 10 min. Cells were then washed three times with PBS 1× 1 mM MgCl_2_ pH 5.7 and incubated with 2 mL X-Gal staining solution (5 mM K_3_Fe(CN)_6_, 5 mM K_4_Fe(CN)_6_)·3H_2_O, 1 mg/mL X-Gal, 150 mM NaCl in PBS 1× 1 mM MgCl_2_ pH 5.7) per well overnight. Plates were washed three times with PBS 1×. Images were acquired with using a DMIL LED Leica microscope (Leica Microsystems, Wetzlar, Germany) and the LAS EZ 3.4 software (Leica Microsystems, Wetzlar, Germany). Quantification was performed using the Fiji ImageJ image analysis software (ImageJ2, version 1.54i, National Institutes of Health, Bethesda, MD, USA).

### 4.3. Lentiviral Infection and Production

IMR90 fibroblasts were infected with the inactive Cas9 vector pLX303-ZIM3-KRAB-dCas9 (Addgene, Watertown, MA, USA), and after selection with blasticidin they were subsequently infected with control (pLenti SpBsmBI sgRNA Puro) or SIRT-targeting guides to target individual sirtuin expression. For the later, the following sequences were cloned into pLenti SpBsmBI sgRNA Puro: SIRT1 (forward guide 5′-ACACCGAGAGGCAGTTGGAAGATGGG-3′ and reverse guide 5′-AAAACCCATCTTCCAACTGCCTCTCG-3′), SIRT2 (forward guide 5′-ACACCGCGCGGTGCTGAAGCCCTTGG-3′ and reverse guide 5′-AAAACCAAGGGCTTCAGCACCGCGCG-3′), SIRT6 (forward guide 5′-ACACCGGCGGAAGCGGCCTCAACAAG-3′ and reverse guide 5′-AAAACTTGTTGAGGCCGCTTCCGCCG-3′), and SIRT7 (forward guide 5′-ACACCGCAGGTCTCCAGGGGAGCGAG-3′ and reverse guide 5′-AAAACTCGCTCCCCTGGAGACCTGCG-3′). For lentiviral production, vectors were cotransfected with psPAX and pMD2G plasmids using polyethylenimine into HEK293T cells. Viral supernatants were collected from HEK293T cells 2 days after transfection and passed through a 0.45 μm syringe filter. The viral supernatant was supplemented with 4 μg/mL polybrene and used to infect IMR90 cells in a 1:10 dilution over a 6 h incubation period. After 2 days, cells were selected with either 4 μg/mL blasticidin or 1 μg/mL puromycin.

### 4.4. Synchrotron-Based Single-Cell FTIR Acquisition Conditions and Spectral Analysis Procedures

For FTIR analysis, cells were cultured on round 12 mm × 0.5 mm CaF_2_ IR polished windows (Crystran Ltd., Dorset, UK) inside 6-well plates. Cells were washed once with PBS 1× and fixed with 4% paraformaldehyde for 20 min. Cells were then washed three times with H_2_O and air-dried before synchrotron analysis. SR-FTIR measurements were performed at the MIRAS beamline of ALBA synchrotron using the Hyperion 3000 microscope (Bruker Optik GmbH, Ettlingen, Germany). coupled to a Vertex 70 spectrometer. The Mercury Cadmium Telluride detector, cooled with liquid nitrogen, was used for spectral collection. The spectra of individual cells (>30 cells/sample) were collected using a mask aperture of 12 × 12 μm^2^, matching the size of a single cell. The infrared spectra were acquired in transmission mode using the 36× objective with 256 scans per spectrum in transmission mode. Acquisition was performed at the spatial resolution of a single cell, with spectra collected from individual, non-overlapping cells. The measurements shown correspond to distinct cells, ensuring that the data reflect cell-to-cell variability. The OPUS 8.2 software package was used for data acquisition. Analysis of spectra was performed using the Quasar 1.10.1 open-source software. Raw spectral data were first baseline-corrected using the rubber band subtraction method to eliminate background interference. Following baseline correction, spectra were normalized using vector normalization to account for differences in overall intensity and enable accurate comparison across samples. For quantification, peak areas were integrated within defined wavelength ranges of previously baseline-corrected and normalize spectra. Second derivative spectra were calculated using a Savitzky–Golay filter with 28 windows to smooth the spectra to enhance subtle spectral features and reduce baseline interference. Second derivative spectra were only used for hidden peak allocation and to investigate peak shifts, always after vector normalization.

### 4.5. RNA Isolation and Quantitative PCR Was Performed

Total RNA was extracted using the Promega Maxwell RNA purification kit (Promega Corporation, Madison, WI, USA) and cDNA was generated using the Transcriptor First Strand cDNA Synthesis Kit. (Roche Diagnostics, Mannheim, Germany). Quantitative PCR was performed with SYBR Green and the QuantStudio quantitative PCR system (Applied Biosystems (Thermo Fisher Scientific), Waltham, MA, USA). Primer sequences used are as follows: ACTB-F 5′-CATGTACGTTGCTATCCAGGC-3′ and ACTB-R 5′-CTCCTTAATGTCACGCACGAT-3′, SIRT1-F 5′-GCCTTGCTGTAGACTTCCCA-3′ and SIRT1-R 5′-CTTTGGATTCCCGCAACCTG-3′; SIRT2-F 5′-CGACTTTCGCTCTCCATCCA-3′ and SIRT2-R 5′-GATGGTTGGCTTGAACTGCC-3′; SIRT6-F 5′-GCAGTCTTCCAGTGTGGTGT-3′; SIRT6-R 5′-AAGGTGGTGTCGAACTTGGG-3′; SIRT7-F 5′-ATTGTGCACTTTGGGGAGAG-3′; SIRT7-R 5′-CAGAGGCGTGGGTACTTCTT-3′.

### 4.6. Genomic DNA Extraction

Cells were washed in PBS 1× and incubated O/N win lysis buffer (1 mM EDTA pH 8, 10 mM NaCl, 1% SDS, 50 mM Tris-HCl, 50 μg/mL proteinase K) at 55 °C. The following day, 400 μL of NaCl 4 M was added to the sample. Samples were incubated 10 min at room temperature and centrifuged for 20 min at 16,000× *g*. Supernatants we transferred to new tubes, and 450 μL of absolute isopropanol was added. Samples were centrifuged for 10 min at 16,000 and supernatants were carefully discarded. Then, two consecutive washes with 70% ethanol were performed. Samples were diluted in H_2_O and quantified using a Nanodrop One device (Thermo Fisher Scientific, Waltham, MA, USA).

### 4.7. Protein Extraction

Cells were washed in PBS 1× and lysed in RIPA buffer supplemented with Protease Inhibitor Cocktail III (Merck KGaA, Darmstadt, Germany) 1:1000 for 15 min on ice and centrifuged at 13,300 rpm at 4 °C. Supernatants were transferred to new tubes. Absorbance was measured at 562 nm. Protein concentration was determined using the Pierce BCA Protein Assay Kit (Thermo Fisher Scientific, Waltham, MA, USA) according to the manufacturer’s protocol, using a standard curve generated with serial dilutions of bovine serum albumin.

### 4.8. Statistical Analysis

Statistical analyses were performed using GraphPad Prism 10 (GraphPad Software, San Diego, CA, USA). Data are presented as mean ± standard error of the mean (SEM). One-way analysis of variance (ANOVA) was used to assess differences between groups. Significance levels are indicated as follows: *p* < 0.05 (*), *p* < 0.01 (**), *p* < 0.001 (***), and *p* < 0.0001 (****).

## 5. Conclusions

Using single-cell SR-FTIR, we have been able to identify changes in the biomolecular structure and composition of senescent cells, which suggests a general metabolic rewiring upon stress induction. Of note, several of these changes were reversed upon treatment with resveratrol, including measurements associated with lipid metabolism, RNA structural conformation, Z-DNA abundance, and DNA/protein abundance ratios, which may suggest a direct role for sirtuins in the acquisition of these features. Further research conducted in other senescent cell types and under different stimuli will be necessary to confirm whether the identified biomolecular changes in this study also occur in a broader context, including during in vivo accumulation of senescent cells in organismal ageing or in tissues from age-related diseases. Nonetheless, the array of biomolecular footprints reported in this study may represent an important step forward in understanding and detecting the global macromolecular alterations associated with the acquisition of the senescent phenotype, which might potentially contribute to the detection of senescent cells, especially in limited abundance samples using single-cell SR-FTIR.

## Data Availability

The original contributions presented in this study are included in the article. Further inquiries can be directed to the corresponding authors.
